# Identification of a proton sensor that regulates conductance and open time of single hERG channels

**DOI:** 10.1038/s41598-019-56081-y

**Published:** 2019-12-27

**Authors:** Stacey L. Wilson, Christopher E. Dempsey, Jules C. Hancox, Neil V. Marrion

**Affiliations:** 1 School of Physiology, Pharmacology & Neuroscience, University of Bristol, Bristol, BS8 1TD UK; 2 School of Biochemistry, University of Bristol, Bristol, BS8 1TD UK; 3 Present Address: Covance, Wooley Road, Alconbury, Huntingdon, Cambridgeshire PE28 4HS UK

**Keywords:** Biophysics, Physiology, Structural biology, Cardiology

## Abstract

The hERG potassium channel influences ventricular action potential duration. Extracellular acidosis occurs in pathological states including cardiac ischaemia. It reduces the amplitude of hERG current and speeds up deactivation, which can alter cardiac excitability. This study aimed to identify the site of action by which extracellular protons regulate the amplitude of macroscopic hERG current. Recordings of macroscopic and single hERG1a and 1b channel activity, mutagenesis, and the recent cryoEM structure for hERG were employed. Single hERG1a and 1b channels displayed open times that decreased with membrane depolarization, suggestive of a blocking mechanism that senses approximately 20% of the membrane electric field. This mechanism was sensitive to pH; extracellular acidosis reduced both hERG1a and1b channel open time and conductance. The effects of acidosis on macroscopic current amplitude and deactivation displayed different sensitivities to protons. Point mutation of a pair of residues (E575/H578) in the pore turret abolished the acidosis-induced decrease of current amplitude, without affecting the change in current deactivation. In single hERG1a channel recordings, the conductance of the double-mutant channel was unaffected by extracellular acidosis. These findings identify residues in the outer turret of the hERG channel that act as a proton sensor to regulate open time and channel conductance.

## Introduction

Cardiac ventricular action potential duration is significantly influenced by the activity of the hERG potassium channel (encoded by *human-Ether-à-go-go Related Gene*; alternative nomenclature *KCNH2*) that underlies the native “I_Kr_” current^1,2^. The slow activation of the hERG channel coupled with rapid inactivation restricts I_Kr_ current amplitude early during the plateau phase of the action potential, with the rapid recovery from inactivation permitting this current to repolarize the ventricular action potential^3^. hERG activity persists to conduct current early in diastole, which provides the ventricles some protection from premature excitation^4^. Alterations to hERG function are implicated in both inherited and acquired arrhythmias^2,3,5^. Extracellular acidosis occurs during myocardial ischemia and reperfusion and is well recognized to be responsible for changes in myocardial ion handling that can cause arrhythmias^6,7^. It reduces the peak amplitude, shifts the voltage dependence of activation, and accelerates deactivation of I_Kr_/I_hERG_^8–15^. Earlier studies have shown that current amplitude and current deactivation display different sensitivities to changes in proton concentration, reflected in different pK_a_ values and suggesting more than one proton binding site on the hERG channel^8,12,15^.

Cations and protons compete for a binding site within the hERG channel, with an increase in extracellular calcium (Ca^2+^) concentration inhibiting the shift in activation evoked by an increase in extracellular protons^16^. In contrast, raising extracellular Ca^2+^ did not affect the reduction in current amplitude evoked by reducing extracellular pH (pH_e_)^11^. Mutagenesis studies have confirmed that residues in S2 (D456 and D460) and S3 (D509) transmembrane helices of the hERG voltage sensor domain bind cations^16–18^, with mutation of all three residues abolishing the shift in activation but retaining the reduction in current amplitude and acceleration of deactivation evoked by pH_e_^18^. Both acidic pH_e_ and neutralization of D509 destabilize voltage-sensor relaxation and shift the voltage dependence of hERG deactivation^19^. These data strongly support the presence of multiple binding sites for protons, with the residues imparting effects of acidosis on hERG current amplitude as yet unknown.

It is crucial to identify residues that affect reduction of hERG current amplitude, in order then to be able to determine whether this effect is modifiable and potentially therapeutically tractable. Determination of the sensitivity of different features of hERG current (I_hERG_) to extracellular proton concentration has suggested that histidine residues are implicated^8,9,12^. However, mutation of likely histidine residues failed to reveal how the amplitude and deactivation of hERG-mediated current are affected by pH_e_^10,15^. Moreover, it is not known whether the entire effect of extracellular acidosis on macroscopic current amplitude is attributable to reduction of single channel current amplitude^15^. We have used a multidisciplinary approach to determine how hERG channel current magnitude is affected by extracellular acidosis. Acidic pH_e_ reduced macroscopic current amplitude and accelerated deactivation kinetics with a pH sensitivity that implies a role for histidine residues. An increase of extracellular protons reduced the conductance and open time of single hERG1a and 1b channels, suggesting that channel opening is curtailed by a proton-sensitive mechanism. Mutagenesis of targeted histidine residues failed to identify any involved in the effects of acidosis on current amplitude or kinetics. However, a double mutation within the outer turret of the channel pore did prevent the effect of pH_e_ reduction on macroscopic current amplitude, but not on deactivation kinetics. Single channel conductance and open time of the double mutant hERG channel was not affected by changes in pH_e_. These data reveal that hERG channels are novel in utilising an outer turret amino acid motif that connects to a mechanism to regulate conductance and open time.

## Results

### Extracellular acidosis inhibits macroscopic hERG current

**Figure 1:**
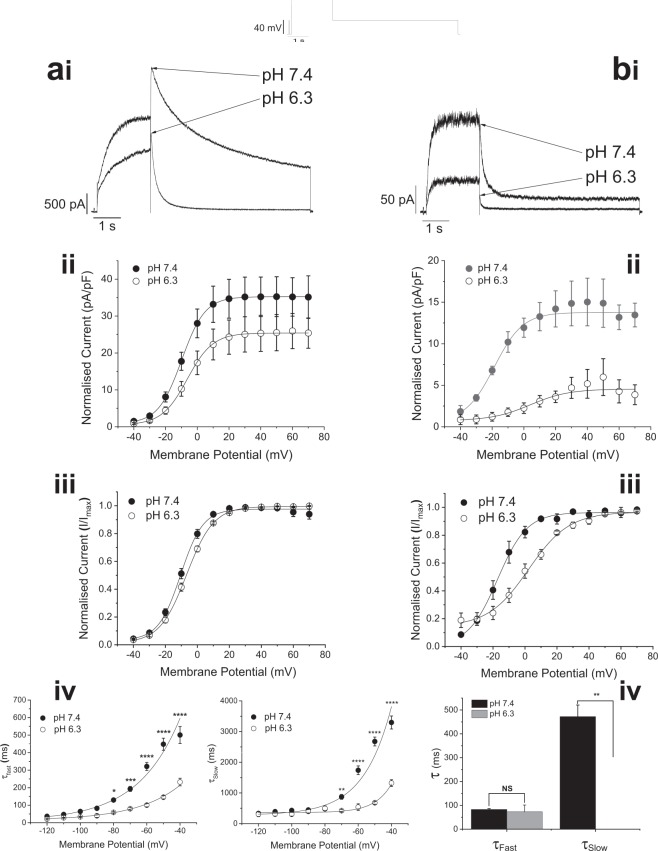
hERG1a- and 1b-mediated current is inhibited by extracellular acidosis. (**ai**) HERG1a-mediated current evoked by the protocol in inset is reduced in amplitude and current deactivation is accelerated by addition of extracellular solution with a pH of 6.3. (**aii** and **iii**) Extracellular acidification causes a shift in voltage-dependence of activation of hERG1a-mediated current, with V_0.5_ of −10.0 ± 1.2 mV (k = 6.8 ± 0.2 mV) at pH 7.4 (n = 15 cells) shifted to a V_0.5_ of −7.0 ± 2.8 mV (k = 8.3 ± 1.1 mV) at pH 6.3 (n = 8 cells)**. (aii**) Shows mean current-voltage (I–V) relations for I_hERG_ tails at pH 7.4 and 6.3, expressed in pA/pF, whilst in (**aiii**) currents following each test potential were normalized to the maximum current (I_max_) elicited by the protocol. (**aiv**) Graphs showing acceleration by acidic pH_e_ of the two time -course components of hERG1a-mediated current with hyperpolarization. Data are shown as mean ± S.E.M for the fast time constant (τ_Fast_) (left) at pH 7.4 (•)(n = 15 cells) and 6.3 (○)(n = 8 cells) and slow time constant (τ_Slow_) (right) of deactivation at pH 7.4 (•)(n = 15 cells) and 6.3 (○)(n = 8 cells). The fast component of current deactivation changed e-fold in 26 mV. Application of pH 6.3 accelerated the fast time component without affecting apparent voltage dependence. In contrast, the slow component of deactivation changed e-fold in 20 mV, with pH 6.3 solution increasing the rate of this component and reducing the apparent voltage dependence to e-fold in 30 mV. ‘*’, ‘**’, ‘***’, and ‘****’ denote statistical significance of P < 0.05, P < 0.01, P < 0.001 and P < 0.0001 respectively (2-way ANOVA with Bonferroni post-test). (**bi**) hERG1b-mediated current (same protocol as hERG1a) was reduced in amplitude and current deactivation is accelerated by addition of extracellular solution with a pH of 6.3. (**bii** and **iii**) The voltage dependence of activation of hERG1b-mediated current was significantly positively shifted, with the V_0.5_ value changing from −16.2 ± 2.8 mV (k = 7.6 ± 0.6 mV) at pH 7.4 (n = 7 cells) to a V_0.5_ of +2.2 ± 1.1 mV (k = 12.6 ± 1.0 mV) at pH 6.3 (n = 7 cells). (**bii**) Shows mean current-voltage (I–V) relations for I_hERG_ tails at pH 7.4 and 6.3, expressed in pA/pF, whilst in (**biii**) currents following each test potential were normalized to the maximum current (I_max_) elicited by the protocol. (**biv**) The time-course of the deactivation of hERG1b-mediated current was accelerated by reduction of pH_e_. The bar chart shows mean ± SEM data for the fast (τ_Fast_) and slow (τ_Slow_) time constant components of hERG1b deactivation measured upon repolarization to −40 mV. Addition of pH 6.3 solution did not affect τ_Fast_ (P = 0.7469; paired t-test), but significantly accelerated τ_slow_ (P = 0.0074; paired *t*-test).

Extracellular pH can be as low as 6.3 during acidosis following cardiac ischemia^20^. Application of a pH 6.3 extracellular solution inhibited expressed hERG1a or 1b-mediated current. Current was evoked by membrane depolarization from a hyperpolarized holding potential (−80 mV), revealing the enhanced outward tail current resulting from the rapid recovery from inactivation induced by membrane repolarization^1,2^. The macroscopic hERG conductance was reduced by extracellular acidosis, with maximal conductance (G_max_) of hERG1a current reduced by 29 ± 0.9% (n = 8, P = 0.0003) and hERG1b current by 69 ± 3.5% (n = 7, P < 0.0001). The amplitude of hERG1a (Fig. 1ai) or hERG1b (Fig. 1bi) current was reduced throughout the voltage range and the voltage-dependence of activation was shifted depolarized in the presence of pH 6.3 solution (Fig. 1aii,aiii for hERG1a, and Fig. 1 bii,biii for hERG1b). Voltage dependence of hERG1a activation (V_0.5_ of −10.0 ± 1.2 mV (k = 6.8 ± 0.2 mV) at pH 7.4 (n = 15 cells) shifted slightly to a V_0.5_ of −7.0 ± 2.8 mV (k = 8.3 ± 1.1 mV) at pH 6.3 (n = 8 cells; (Fig. 1aiii) and at pH 5.5 was further shifted to V_0.5_ of + 11.8 ± 2.4 mV (k = 11.9 ± 0.5 mV; n = 7 cells; not shown; p < 0.001 for V_0.5_; ANOVA). This is consistent with prior studies reporting rightward shifted activation of I_hERG_/I_Kr_ at acidic pH_e_^11,14,19,21^. Reduction of hERG1b-mediated current at acidic pH was accompanied by a more marked shift in voltage dependence of activation (V_0.5_ −16.2 ± 2.8 mV (k = 7.6 ± 0.6 mV) at pH 7.4 (n = 7 cells) to a V_0.5_ of + 2.2 ± 1.1 mV (k = 12.6 ± 1.0 mV) on changing to pH 6.3 (n = 7 cells) (Fig. 1biii; p < 0.001 for V_0.5_ and p < 0.01 k; *t-*test)). Due to the difficulty in recording macroscopic current from cells transfected with hERG1b alone, a lower extracellular pH than 6.3 was not applied in hERG1b experiments. Acidic pH_e_ accelerated the macroscopic current deactivation rate. hERG1a current deactivation was best described by a bi-exponential time-course that accelerates with hyperpolarization (Fig. 1aiv). Both time components became significantly faster upon extracellular acidification (P < 0.0001; two-way ANOVA with Bonferroni post-test), with a greater acceleration of the slow time component (Fig. 1aiv). The proportion of deactivating current described by the fast time-constant also increased at acidic pH_e_, at potentials positive to −90 mV (Supplementary Fig. S1a) In contrast, only the slow component of hERG1b current deactivation was clearly accelerated by acidic pH_e_ (Fig. 1biv), whilst the proportion of fast/slow deactivating current did not change (Supplementary Fig. S1b).

### Increasing extracellular protons decreases single hERG channel conductance

**Figure 2:**
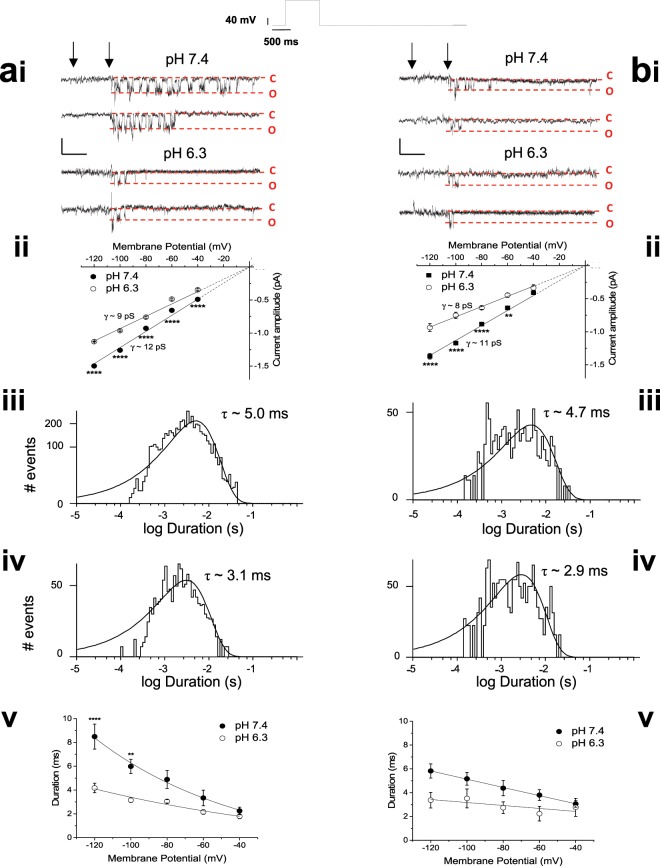
Extracellular acidosis reduces single hERG1a and 1b channel conductance. (**ai** and **bi**). Representative traces of single hERG1a (**bi**) and hERG1b (**bi**) channels at -80 mV, originating from recording with pipette solution pH of 7.4 or pH 6.3. Channel activity was observed following a voltage step to +20 mV, imposed as indicated by the arrows. Openings are shown as downward deflections, with dashed lines to represent the closed (C) and open (O) state. (**ai**) Single channel openings from two patches containing hERG1a channels, one with an electrode solution of pH 7.4 (upper 2 traces, from a single recording) and another with an electrode solution of pH 6.3 (lower 2 traces, from a single recording). (**bi**) hERG1b channel openings from two patches, one with an electrode solution of pH 7.4 (upper 2 traces from a single recording) and another with an electrode solution of pH 6.3 (lower 2 traces from a single recording). Horizontal scale bar represents 500 ms and vertical scale bar represents 1 pA. Single-channel current-voltage (I-V) relationships for hERG1a (**aii**) and hERG1b (**bii**) channels at the two pH values. Channel amplitudes were derived from amplitude histograms fitted with a single Gaussian distribution (not shown). Plots were fitted with a linear relationship (solid line) constrained to pass through zero (dashed line) to achieve slope conductance 12.3 ± 0.2 pS for pH 7.4 (n = 10 cells) and 9.3 ± 0.1 pS for pH 6.3 (n = 9 cells; P < 0.0001, unpaired students t-test) for hERG1a channels (**aii**). Slope conductance values for hERG1b channels (**bii**) are 11.3 ± 0.2 pS for pH 7.4 (n = 7 cells) and 7.7 ± 0.4 pS for pH 6.3 (n = 6 cells; P < 0.0001, unpaired students t-test). SEM bars are small and obscured by the symbols for some points. (**aiii** and **biii**) Open-time duration histograms produced at -80 mV by combining data from 10 patches in pH 7.4 for hERG1a (aiii) and 7 patches for hERG1b (biii). Histograms were fit with a single exponential function with time constants of 5.0 ms (hERG1a) and 4.7 ms (hERG1b). (**aiv** and **Biv**) Combined open-time duration histograms for 9 patches in pH 6.3 at -80 mV for hERG1a (**aiv**) and hERG1b (**biv**). Histograms were fit with a single exponential function with time constants 3.1 ms (hERG1a) and 2.9 ms (hERG1b). (**av** and **bv**) Plots of open time vs membrane potential for hERG1a (**av**) and hERG1b (**bv**) channels, with exponential fits at 7.4 and pH 6.3. Channel open time decreased with membrane depolarization, with the voltage dependence of the change in open time with voltage being lost in pH 6.3. ‘****’ and ‘**’ denote significance of P < 0.0001 and P < 0.01 respectively (2-way ANOVA).

The observed reductions in macroscopic hERG conductance can derive from changes in single channel conductance, open time and/or a reduction in the number of functional channels. The effect of acidic pH_e_ was rapidly reversible (data not shown), which suggests that protons likely affect channel conductance^15^ or open time. Prior single channel data on effects of acidosis are restricted to evidence that single hERG channel current amplitude of the attenuated inactivation S620T channel is reduced at pH 5.5 compared with 7.4^15^. Figure 2ai,bi respectively show example recordings of hERG1a and 1b single channel currents at pH_e_ of 7.4 and 6.3. Single hERG1a channels exhibited a slope conductance of 12.2 ± 0.1 pS at extracellular pH 7.4 (n = 9 patches) (Fig. 2aii). In contrast, single hERG1b channels exhibited a slope conductance of 11.3 ± 0.2 pS under identical conditions (n = 7 patches) (Fig. 2bii). To our knowledge, these are the first single channel data reported for the hERG1b isoform. The greater reduction in macroscopic hERG1b-mediated current amplitude by reduction of pH_e_ is partially reflected in the effects on single channel conductance. Single channel conductance was reduced by extracellular pH 6.3, with the slope conductance of hERG1a channels reduced from 12.2 pS in pH 7.4 to 9.3 ± 0.1 pS in pH 6.3 (n = 9; P < 0.0001, unpaired Students t-test) (Fig. 2aii). This 24% reduction in single channel conductance is comparable with the 29% reduction in the macroscopic G_max_. In contrast, the single hERG1b conductance was reduced from 11.3 pS in pH 7.4 to 7.7 ± 0.4 pS in pH 6.3 (n = 6)(P < 0.0001; unpaired *t*-test) (Fig. 2bii). This 32% reduction in single channel conductance partially contributes to the 69% reduction in G_max_.

Extracellular acidosis must affect other single channel properties to produce the observed reduction in macroscopic conductance (G_max_). Single hERG1a and 1b channels exhibited a single open state (Fig. 2aiii,biii). Both hERG1a and 1b channel open time decreased with membrane depolarization^22^. Channel mean open time for both homomeric channels was reduced by acidic pH_e_. For example, pH 6.3 caused hERG1a channel open time to decrease from a mean open time of 5.0 ms in pH 7.4 (Fig. 2aiii), to 3.1 ms in pH 6.3 (Fig. 2aiv) at −80 mV. A similar reduction in mean open time was seen with hERG1b channels, where mean open time of 4.7 ms in pH 7.4 (Fig. 2biii) was reduced to 2.9 ms in pH 6.3 at −80 mV (Fig. 2biv). In addition, the apparent voltage dependence of open time for both homomeric channels was considerably reduced in acidic extracellular pH (Fig. 2av,bv).

**Figure 3:**
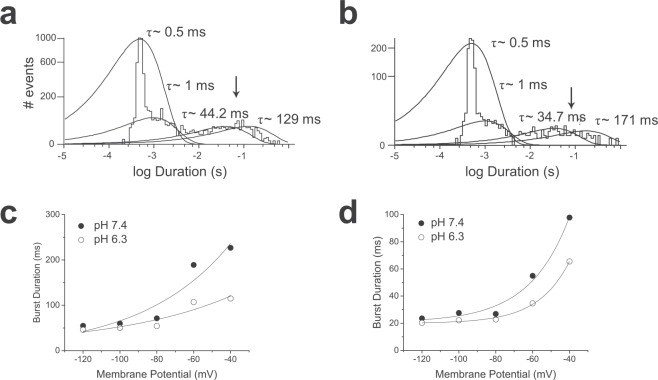
Extracellular acidosis shortens hERG1 channel burst duration. (**a**,**b)** Closed-time distributions for hERG1a (**a**) and hERG1b (**b**) in pH 7.4 at -80 mV. Distributions for both channel isoforms were best described by four exponentials with time constants (% contribution) of 0.48 ms (89.2%), 0.98 ms (5.7%), 44.2 ms (2.5%) and 129 ms (2.6%) for hERG1a. The closed time distribution for hERG1b was 0.51 ms (86.3%), 0.86 ms (8.1%), 20.5 ms (3.8%) and 384 ms (1.8%). τ_crit_ values were calculated between the third and fourth component of closed-time distributions as indicated by an arrow. (**c**) Burst durations of hERG1a channels were obtained from histograms comprising of 10 patches for pH 7.4 and 9 patches for pH 6.3 and plotted against the respective membrane potential. The plot was fit with an exponential function, showing the hERG1a channel burst duration accelerates e-fold in 40 mV. This voltage-dependence is largely lost in pH 6.3. (**d**) hERG1b burst durations obtained from histograms comprising of 7 patches for pH 7.4 and 6 patches for pH 6.3. Data were fitted with an exponential function, showing the hERG1b channel burst duration accelerates e-fold in 30 mV, a voltage dependence retained at pH 6.3.

Single hERG1a (Fig. 3a) and 1b (Fig. 3b) channels exhibit a closed-time distribution that was best described by the sum of four exponentials in pH 7.4. A rapid closed time distribution of time constant of approximately 0.5 ms was resolved, which likely reflects rapid closures within a burst. Burst duration for both hERG1a and 1b channels showed voltage dependence, with shorter burst durations observed with membrane hyperpolarization (Fig. 3c,d), which is reflected in the voltage dependence of macroscopic current deactivation. Lowered pH_e_ reduced burst duration, but it had little effect on apparent voltage dependence (Fig. 3c,d).

### Proton sensitivity of hERG channel gating

**Figure 4:**
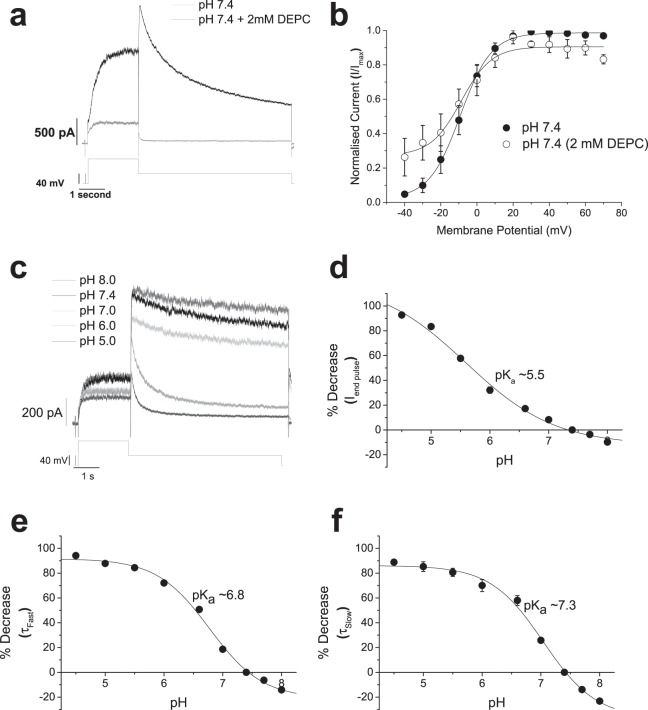
A possible role of histidine residues in the effect of extracellular acidosis. (**a**) Representative hERG1a currents in control (black trace) and after 10 minutes of 2 mM DEPC application (grey trace), showing a significant reduction in the amplitude and acceleration of current deactivation by the histidine residue modifier. (**b**) Steady-state activation plots from hERG1a I_Tail_ upon repolarisation to −40 mV. For control and 2 mM DEPC, values were normalized to maximal I_Tail_ in each condition and plotted against membrane potential. Relations were fit with a Boltzmann function to give V_0.5_ values of −9.1 ± 3.2 mV (k = 7.1 ± 0.4 mV) in control (n = 7 cells) and a V_0.5_ of −4.2 ± 4.2 mV (k = 8.0 ± 1.9 mV) with 2 mM DEPC treatment (n = 7 cells). (**c**) Representative current traces of hERG1a current when subjected to extracellular solutions with pH values decreasing from 8.0 to 5.0. (**d**) Concentration-inhibition curve for reduction of hERG1a current evoked by a step from −80 mV to +20 mV by decreasing extracellular pH. Data are shown as mean ± SEM and fit with the Hill equation to derive a pK_a_ value of 5.5 ± 0.2 with a slope (k) of −0.6 ± 0.1 (n = 7). (**e**,**f**) hERG1a tail current deactivation was fit with a bi-exponential function to achieve τ_Fast_ (**e**) and τ_Slow_ (**f**). Plots show mean ± SEM values of % decrease in τ_Fast_ (**e**) and τ_Slow_ (**f**) against respective pH_e_ value. pK_a_ values derived from fitting the plots with dose-response equation with a variable Hill slope were 6.8 ± 0.1 (k = −1.0 ± 0.1) for τ_Fast_ and 7.3 ± 0.2 (k = −1.0 ± 0.2) for τ_Slow_.

Covalent modification of histidine residues in proteins by diethylpyrocarbonate (DEPC), which removes the histidine residue’s ability to be reversibly protonated, had produced conflicting results when applied to hERG channel current^10,23^. Application of DEPC (2 mM) reduced macroscopic hERG1a current and increased the rate of current deactivation (Fig. 4a), without shifting the voltage-dependence of activation (Fig. 4b). These data are consistent with the lack of histidine residues being involved in the voltage dependence of activation^18^ and suggest that effects on current amplitude and deactivation rate involve histidine residues.

Application of different pH external solutions to hERG1a-mediated macroscopic current permitted the sensitivity to protons of current amplitude and kinetics to be assessed. Figure 4c shows macroscopic hERG1a-mediated current in the presence of extracellular solutions of different pH values. Concentration-response relationships were constructed for effects of proton concentration on macroscopic conductance and deactivation kinetics and are plotted in Fig. 4d–f. Macroscopic conductance and time-course of current deactivation were progressively affected by pH_e_ to give pK_a_ values of 5.5 ± 0.2 (n = 7) for reduction in macroscopic current amplitude (Fig. 4d), 6.8 ± 0.1 for acceleration of the fast component of deactivation (Fig. 4e), and 7.3 ± 0.2 (n = 7) for the acceleration of the slow component of current deactivation (Fig. 4f). These latter two values were not significantly different from each other (P = 0.1065). It is worth noting that the slope of relationships of the effect of pH_e_ on the fast and slow components of deactivation was approximately −1. In contrast, the relationship of inhibition of current amplitude by protons displayed a slope of −0.6 ± 0.1 (Fig. 4d). These data suggest that protons bind to one site to accelerate deactivation but bind to two or more sites that exhibit negative cooperativity to inhibit macroscopic current.

### Point mutation of candidate amino acid residues does not reduce the inhibitory effect of acidic pH_e_

**Figure 5:**
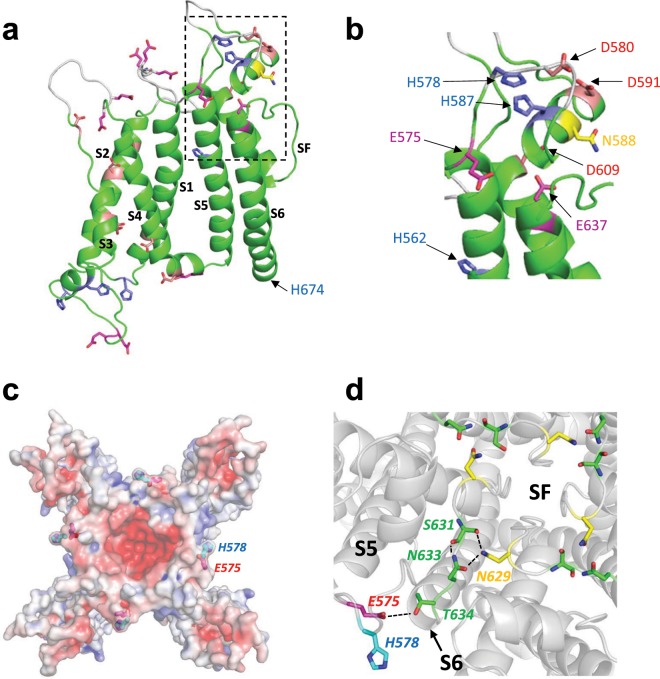
Structural context of target titratable residues. (**a**) Single subunit of the cryo-EM structure of the hERG1a channel^24^ with transmembrane domains labelled. Regions of extracellular loops colored white are absent in the cryoEM structure (PDB:5VA1)^24^ and were modelled into the structure using Modeller v9.17^34^ (https://salilab.org/modeller/). The region enclosed in the dashed box comprises most of the extracellular turret between helices S5 and S6, and is expanded in (**b**). (**c**) Location of the E575/H578 pair mapped onto the electrostatic surface (at pH 7.4; 0.15 mM NaCl) on the extracellular side of the hERG1a channel. (**d**) The E575/H578 motif is linked via an E575 (on S5) –T634 (S6) charge/hydrogen bond interaction to a hydrogen-bonded network involving N633, S631 with N629 on adjacent subunits of the hERG1a channel that forms a ring around the top of the selectivity filter (SF).

**Figure 6:**
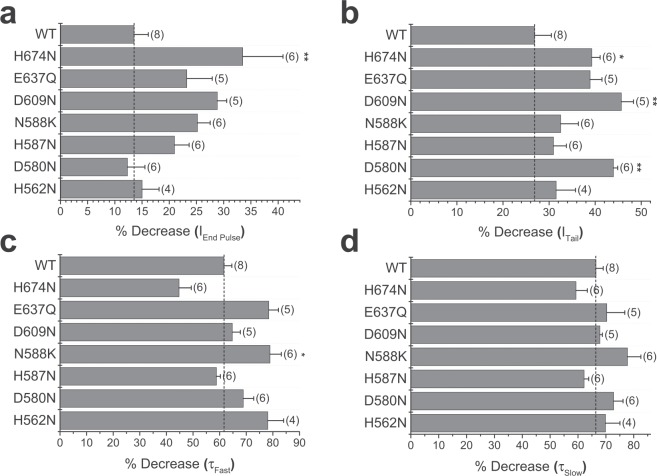
Extracellular acidosis affects hERG1a single point mutants. The effects of extracellular acidosis on the wildtype (WT) hERG1a and channels derived with single point mutations. The vertical dashed line represents the effect seen on WT hERG1a channels. (**a**–**d)** The effects of extracellular acidosis on WT and single point mutations. % decrease of hERG channel characteristics were observed: I_End Pulse_ (**a**), I_Tail_ (**b**), and the fast (τ_Fast_, **c**) and slow (τ_Slow_, **d**) components of deactivation. The data derive from I_hERG_ measured using depolarizing voltage commands to +20 mV, followed by repolarization to −40 mV. Example current traces are included in Supplementary Fig. 2. Numbers in brackets indicate cell numbers. * and ** denote statistical significance of P < 0.01 and P < 0.001 respectively, when compared with values obtained from WT channel current (one-way ANOVA with Bonferroni post-test).

Resolution of a structure for hERG by cryoEM^24^ has allowed an indication of which titratable residues are accessible from the extracellular surface of the channel (Fig. 5a). Previously, single point mutations of candidate histidine residues did not affect the pH sensitivity of hERG1a^15^, with the double mutation of H578 and H587 also failing to affect sensitivity of hERG1a to pH_e_^10^. There is a cluster of titratable residues located at the top of the S5 and S6 region including the S5-P linker or outer turret and the pore helix (Fig. 5a dashed box & Fig. 5b). The following conservative mutations were produced (H562N, D580N, H587N, D609N, E637Q, H674N). The inactivation-impairing mutation N588K was also tested^25,26^. All mutants were tested as current derived from homomeric channels transiently expressed in HEK293 cells, except the H562N mutant. Expression of this construct failed to give functional current, suggesting that the mutation had disrupted trafficking. In this instance, the H562N channel subunits were co-expressed with wildtype hERG1a channel subunits to form heteromeric channels. Figure 6 shows summary bar charts of the effects of pH 6.3 on macroscopic current amplitude, and the fast and slow components of current deactivation for the listed mutants. Representative macroscopic currents from each mutant are shown at each pH in Supplementary Fig. 2. No mutation affected the ability or the magnitude of the effect of protons to *reduce* current amplitude or *accelerate* deactivation. In addition, no mutant affected the shift in voltage-dependence of activation produced by extracellular acidosis (data not shown). Some mutations (H674N, D609N and D580N) were associated with *increased* mean responses of I_Tail_ and/or I_End Pulse_ to pH 6.3 (Fig. 6a,b). Whilst the effect of H674N, which is located intracellularly, is unlikely to reflect a direct interaction with external protons, these observations were not pursued in further detail as this lay outside the intended scope of the present study, which sought to identify residues obligatory for the *inhibitory* proton effect.

### Double mutation within the outer turret prevents proton-mediated reduction of current

**Figure 7:**
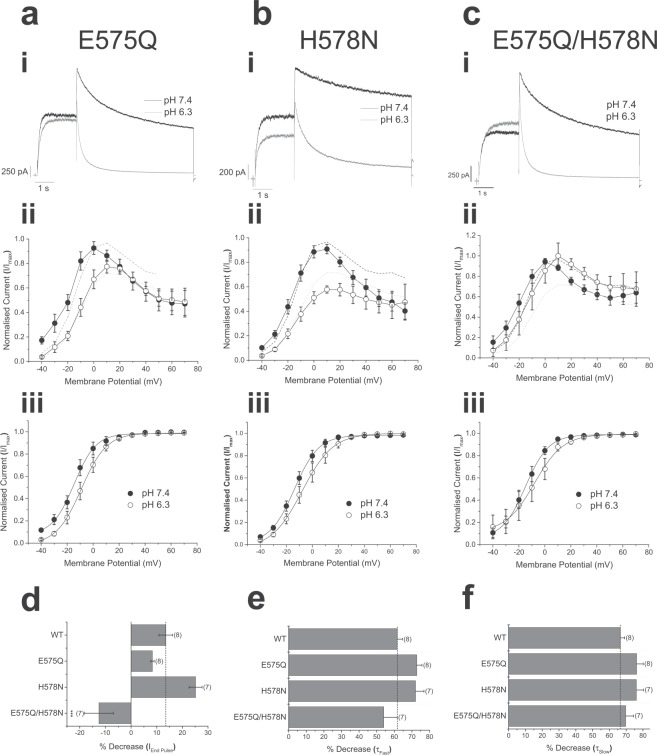
Effects of extracellular acidosis on current amplitude abolished by double mutation of E575 and H578. (**ai-ci**) Membrane current traces of single point mutant (**ai** and **bi**) hERG1a channel currents and the double mutant E575Q/H578N, evoked by step depolarization to +20 mV and repolarization to −40 mV (holding potential −80 mV). Application of pH 6.3 to hERG1a-E575Q had little effect on macroscopic current amplitude positive to +20 mV, but significantly accelerated current deactivation. Greater inhibition of macroscopic current by pH 6.3 of current mediated by hERG1a-H578N was observed, with less effect on the time-course of current deactivation. In contrast, hERG1a-E575Q/H578N-mediated current was augmented by extracellular acidosis at +20 mV, with a clear acceleration of current deactivation. (**aii-bii**) Current-voltage relationships for the two single point mutants and the double mutant of hERG1a channels. Extracellular acidosis reduced the amplitude of evoked current at potentials negative to +20 mV, with little effect at more depolarized levels. In contrast, current amplitude mediated by hERG1a-E575Q/H578N was unaffected at potentials negative to +20 mV, but was augmented at more depolarized levels. The normalized current-voltage relationship for wildtype hERG1a current is shown on each plot as superimposed dashed lines (black, pH 7.4; grey, pH 6.3). (**aiii-ciii**) Activation curves showing that each single and double mutant hERG1a channel current was affected by application of pH 6.3 extracellular solution, with voltage dependence of activation being shifted positive by acidosis. (**d**–**f**) Bar charts showing the effect of extracellular acidosis on WT, hERG1a-E575Q, hERG1a-H578N and hERG1a-E575Q/H578N on the amplitude of the end pulse current evoked by a step to +20 mV (VH −80 mV) (**d**), the fast time component (τ_Fast_) (**e**), and slow time component (τ_Slow_) (**f**), of current deactivation. Numbers in brackets indicate cell replicates.

Residues E575 and H578 lie near to each other on the same loop at the top of the S5 helix (Fig. 5a,b), and backbone flexibility in the loop between the top of S5 and the turret helix may allow close approaches of their side chains (Supplementary Fig. 3). The mutants E575Q and H578N were assessed separately for sensitivity to pH_e_. Both single mutations produced currents that were sensitive to pH 6.3 extracellular solution, with current amplitude reduced, voltage-dependence of activation shifted depolarized, and deactivation of macroscopic current accelerated (Fig. 7a,b,d–f). The effect of protons on both single point mutants was like those seen on WT hERG1a current, except that proton block was not apparent during voltage steps to depolarized potentials (Fig. 7aii,bii). These data suggest that each residue might contribute to the effect of protons. Acidic pH_e_-mediated reduction in current amplitude displayed a relationship that suggests that two or more sites are required for proton binding (Fig. 4d). The acidic pK_a_ of 5.5 for reduction in current amplitude might be an intermediate value arising from the involvement of two residues as binding sites, one with an acidic pK_a_ (calculated pK_a_ value of 4.8 for E575) and another with a more basic pK_a_ (calculated pK_a_ value of 6.3 for H578). Both E575 and H578 are positioned on the external surface of the hERG channel (Fig. 5) and these ionisable side chains groups will most likely display pK_a_ values affected by charge-charge interactions^27,28^.

Extracellular acidosis had a different effect on hERG1a current mediated by the E575Q/H578N double mutant. Application of pH 6.3 extracellular solution had no significant effect on current amplitude over negative test potentials but enhanced current amplitude during pulses to depolarized test potentials (Fig. 7ci,cii,d). The double mutation significantly reduced inhibition of tail current amplitude by pH_e_ 6.3; exposure to this pH_e_ only produced a 11.1 ± 4.0% (n = 7) reduction compared with the 26.8 ± 3.7% inhibition of wildtype hERG1a-mediated current (P = 0.0083; one-way ANOVA with Bonferroni post-test) (Fig. 7ci). However, a reduction of pH_e_ did hasten current deactivation and shift the voltage-dependence of activation as seen for WT channels (Fig. 7ci,iii,e,f). The profile of currents shown in Fig. 7ci and the retention of the negative slope region at positive potentials in the end-pulse current I-V relation (Fig. 7cii), suggest that the double mutation E575Q/H578N did not impair voltage-dependent inactivation of I_hERG_. Additionally, the ability of protons to decrease I_hERG_ amplitude is known not to require intact inactivation (^15,29^). This is supported by the effect of acidic pH_e_ on the inactivation-impaired N588K mutant of hERG1a (Fig. 6 and Supplemental Fig. 2). It is clear that the E575Q/H578N mutation impairs the ability of protons to decrease I_hERG_ conductance. However, the most direct demonstration of this would derive from single channel recording.

**Figure 8:**
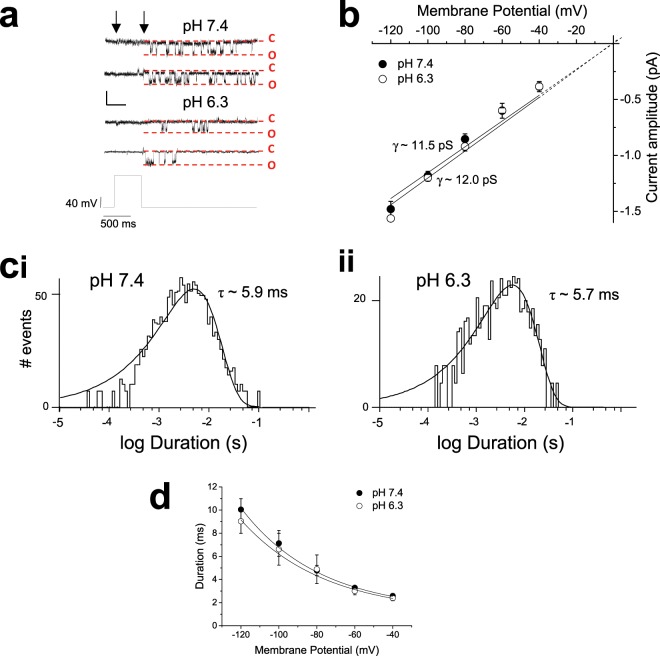
Single channel conductance of hERG1a E575Q/H578N is resistant to extracellular acidosis. (**a**) Representative traces of single hERG1a E575Q/H578N channels at -80 mV, recorded with a pipette solution pH of 7.4 (upper panel, 2 traces from a single recording) and pH 6.3 (lower panel, 2 traces from a single recording). Channel openings were evoked by a prepulse to +20 mV (V_H_ −80 mV) and observed following repolarization to -80 mV. Horizontal scale bar represents 500 ms and vertical scale bar represents 1 pA. (**b**) Single channel current-voltage relationship of hERG1a E575Q/H578N channels in pH 7.4 (•) and 6.3 (○). Channel conductance was not affected by extracellular acidosis, with a slope conductance of 11.7 ± 0.4 pS for pH 7.4 (n = 5 cells) and 12.0 ± 0.5 pS for pH 6.3 (n = 6 cells; P = 0.7032, unpaired student’s t-test). (**ci**) Open-time duration histogram derived from 5 patches with at pH 7.4. Channel activity at -80 mV was fit with a single exponential function with time constant 5.9 ms. (**cii**). Open duration histogram from 6 patches with at pH 6.3. Channel activity at −80 mV was not affected by acidic pH, being fit with a single exponential component with time constant 5.7 ms. (**d**) The voltage dependence of hERG1a-E575Q/H578N channel open time was not affected by pH_e_. Plots were fit with an exponential function for both pH 7.4 (n = 5 cells) and pH 6.3 (n = 6 cells). No significance was observed between pH and membrane potential (two-way ANOVA with Bonferroni post-test).

Single channel recording from transfected cells expressing the hERG1a E575Q/H578N subunit displayed channel openings (Fig. 8a) with a conductance of 11.7 ± 0.4 pS at pH 7.4 (n = 5) (Fig. 8b). This conductance was not significantly different from the conductance of WT hERG1a channels (P = 0.1280; unpaired student’s t-test). Mutant channels exhibited open times that were very similar to WT channels, with a mean open time of 5.9 ms at −80 mV (as compared with 5.1 ms for WT channels, P = 0.9182, unpaired student’s t-test) (Fig. 8c). As observed with WT hERG1a channel activity, the hERG1a E575Q/H578N double mutant channel open time decreased with membrane depolarization (Fig. 8d). These data show that mutation of the two residues in the channel S5-P linker or pore outer turret had no significant effect upon channel properties. However, the effect of extracellular pH 6.3 solution on single current amplitude was abolished (Fig. 8a). Acidic pH_e_ had no effect on channel conductance (Fig. 8b). Channel conductance in pH 6.3 was 12.0 ± 0.6 pS (n = 6; P = 0.7032; unpaired *t*-test). WT channel open times were curtailed by extracellular acidosis (Fig. 2aiv), while pH 6.3 solution had no effect on open time of the hERG1a E575Q/H578N double mutant channel (Fig. 8c). The deactivation time-course of the macroscopic current evoked by the E575Q/H578N double mutant channel accelerated with pH_e_ (Fig. 7ci), yet single channel open time was not affected (Fig. 8d). This clearly indicates that channel open time does not dictate the time-course for hERG1a current deactivation, but instead is dictated by burst duration. This is supported by the decrease in burst duration observed with the double mutant channel upon extracellular acidification from 157.4 to 103.2 ms (measured at −80 mV, data not shown).

## Discussion

Extracellular acidosis causes a reduction in hERG/I_Kr_ amplitude and accelerates current deactivation^8–15^. Incorporation of these effects of acidosis on hERG/I_Kr_ in a human ventricular action potential model led to a reduction of the contribution of I_Kr_ to ventricular repolarization and to enhanced ventricular excitability^14^. Our data (see Fig. 4d–f) are consistent with previous findings^12,15^ suggestive of multiple binding sites for extracellular protons on hERG: protons affect activation and deactivation processes with distinct pH sensitivities. Furthermore, the identification of mutations that prevent the effect of acidic pH_e_ on I_hERG_ amplitude complements recent data implicating a mechanism for proton-mediated acceleration of I_hERG_ deactivation^19^, which is distinct from that uncovered here for reduced current amplitude.

Resolving single hERG1a and 1b channel activity has provided an insight to how macroscopic hERG current is affected by extracellular acidosis. The voltage-dependence of hERG1a (Fig. 3c) and 1b (Fig. 3d) channel burst duration underlies the acceleration of macroscopic current deactivation with hyperpolarization, as channel open times actually get longer at negative membrane potentials (Fig. 2av,bv). Extracellular acidosis shortens burst duration for both isoforms (Fig. 3c,d), producing a hastening of macroscopic current deactivation. The majority of the reduction of hERG1a macroscopic conductance by extracellular acidosis arose from a reduction in single channel conductance by pH. The modest effect on hERG1a and 1b channel open time by acidic pH suggests that the effect of extracellular protons is not mediated by pore block *per se*. Instead, it is likely that extracellular protons can modulate gating directly. In contrast, the effect of acidic extracellular pH on hERG1b channel current amplitude is more difficult to explain. Macroscopic hERG1b conductance was reduced by 69% (Fig. 1bi), with a hastening of current deactivation. The observation of a greater effect of acidic pH_e_ on hERG1b than hERG1a current amplitude is similar to that reported previously, when greater reductions in I_hERG_ tails and pulse currents at pH_e_ 6.3 were reported for channels containing the hERG1b isoform^29^. Deactivation of hERG1b current is like that seen with hERG1a, albeit faster, in that the time-course of deactivation is governed by burst duration of the single channels. The burst duration of hERG1b channels is shortened by extracellular protons, yielding a hastened deactivation of macroscopic current. The large effect of altering extracellular pH from 7.4 to 6.3 on hERG1b macroscopic conductance cannot be derived from only a 32% reduction in single channel conductance (Fig. 2bii). It was noted that extracellular acidosis has a dramatic effect on the position and slope of the macroscopic current activation relationship (Fig. 1biii). The large reduction in macroscopic hERG1b conductance produced by extracellular acidosis may result from both reduction of single channel conductance and a depolarizing shift of the macroscopic current activation accompanied by a decrease in apparent voltage sensitivity.

The pK_a_ values suggest the role of histidine residues, but individual point mutation of several candidate residues failed to affect sensitivity of hERG1a-mediated current to reduction by protons (Fig. 6a–d)^10,15^. Mutation of either one of two additional residues (E575 and H578) produced a modest effect (Fig. 7a,b). In contrast, low pH_e_ failed to reduce amplitude of current elicited by hERG1a E575Q/H578N double mutant (Fig. 7c), even though the voltage-dependence of activation was still shifted in the depolarized direction ((Fig. 7ciii). The lack of reduction of macroscopic pulse current amplitude was observed with single channels, where single channel conductance and open time of the double mutant was not affected by pH 6.3 (Fig. 8b). These data show that we have identified the sites for proton binding that affect single channel conductance and open time. An additional site is responsible for the increase in rate of macroscopic current deactivation, and therefore burst duration of single channels. This may result from destabilized voltage-sensor relaxation and an altered shift in the voltage dependence of hERG deactivation, as recently described by Shi and colleagues^19^. hERG1a and 1b isoforms differ only in their N termini^30^, thus the E575 and H578 residues are present in hERG1b, represented as E235 and H238. It is likely that the effect of extracellular protons on these residues mediate the observed effects on hERG1b channel conductance. The acidic pH-induced rightward voltage shift to I_hERG_ activation for hERG1a involves residue D509 (in S3), with additional cooperative interactions with D456, D460 (in S2)^18^. These residues are also present in hERG 1b (as D169, D116 and D120, respectively). It is clear however, that the much greater effect of extracellular acidosis (pH 6.3) on hERG1b than hERG1a macroscopic conductance must be contributed either by indirect/allosteric consequences of the unique hERG1b N terminus that favour exposure to protons of these identified sites, or by additional sites that affect the voltage-dependence of activation.

Macroscopic hERG-mediated current is activated by depolarization but the channel displays single channel open times that decrease at positive voltages (Fig. 2av,bv)^22^. The resulting decrease in channel open probability with membrane depolarization is observed with a mutant hERG channel (S631A) that exhibits an extreme depolarizing shift in inactivation^31^, which indicates that this process does not underlie channel inactivation. This behaviour suggests a voltage-dependent process that regulates channel open time, in a manner analogous to channel block. Application of Woodhull analysis shows that open time is curtailed by a process that senses approximately 18% of the membrane field. This process is pH-dependent, with extracellular acidosis collapsing the open time to provide rapid lifetime openings that do not display prominent voltage-dependence (Fig. 2av,bv). The effect of acidic pH_e_ on current amplitude has previously been reported to sense approximately 18% of the membrane field^15^. Mutation of H587 within the turret helix of hERG1a to a glutamate residue changed the configuration of the channel so that the proton binding site only sensed approximately 4% of the membrane field^15^. The effect of glutamate substitution at this site suggests that, as H587 is positioned superficially and would itself not sense approximately 18% of the membrane field (Fig. 5), the effects are transmitted to a site deeper towards the membrane, for example an external region of the selectivity filter. Double mutation of E575/H578 residues abolished the effect of acidic pH_e_, but the position of these residues indicated by the cryoEM structure (Fig. 5) is also too superficial to experience a significant part of the membrane field. In addition, channel open times of the hERG1a E575Q/H578N double mutant decreased with membrane depolarization in a manner similar to wildtype hERG1a channels (cf Figs. 2av and 8d). These data suggest that it is not the E575/H578 amino acid motif itself that senses 18% of the membrane field, but that the conformation of this motif regulates the mechanism that senses the membrane field to regulate the channel open state.

How would the binding of protons to E575 and H578 change the electrostatic environment in and around the selectivity filter? The electrostatic profile of the extracellular surface of the hERG channel shows a concentration of charge around the selectivity filter, but E575 and H578 lie on the outer circumference of the hERG pore domain (Fig. 5c). A hydrogen-bond interaction between a glutamate residue at the top of S5(E575 in hERG) and a threonine residue at the top of S6 (T634 in hERG) is highly conserved amongst all K^+^ channels for which atomic resolution structures have been determined (e.g. MthK, KcsA, KvAP, Kv1.2, hERG and EAG). It is only in hERG that this interaction connects the E575/H578 pair to a hydrogen-bonded network that forms a ring near the outer mouth of the selectivity filter (Fig. 5d). Mutations in this region render the channel poorly expressible (N629A) or cause long QT syndrome through altered gating (N629S, N629D and N633S)^32^. These findings suggest that hERG possesses flexibility of the outer part of the selectivity filter. This flexibility underlies inactivation^24–26^ and provides a mechanism to transmit protonation of E575/H578 to changes in hERG channel conductance and open time. An important qualification in this regard is that in the hERG channel cryo-EM structure, the turret lacks density for residues H578 through R582 and for residues N598 through L602^24^; accordingly, these short loops were modelled into the pore domain structure shown in Fig. 5. There is density, however, for the preceding residue (P577) including the backbone carbonyl carbon, and this provides a good constraint on the location of H578 backbone atoms. As this does not specify the location of the H578 side chains, we have been careful to limit our positional interpretations accordingly, restricting these to the position of the E575 side chain and its interactions with residues that connect to the selectivity filter that are defined in the cryo-EM hERG structure^24^. Future work is warranted to determine the pharmacological tractability of this proton sensor, to ascertain whether or not it may offer a novel route for therapeutic targeting to modulate hERG/I_Kr_ conductance and thereby the channel’s contribution to ventricular repolarization.

## Methods

### Channel expression in HEK293 cells

All recording of wild-type (WT) hERG1a-mediated current and channel activity was accomplished using HEK-293 cells stably expressing WT hERG1a (a kind gift from Dr Craig January, University of Wisconsin)^33^. In contrast, all recordings of hERG1a mutant channels were made from HEK-293 cells transiently transfected with the appropriate construct. Channels mediating native cardiac I_Kr_ may contain both hERG 1a and 1b subunits^30^ and so in this study some experiments were also conducted on hERG1b-mediated current. The hERG1b construct was kindly provided by Dr Gail Robertson (University of Wisconsin)^30^, with hERG1b-mediated currents being recorded from transiently transfected CHO cells^29^. Single and double point mutations were introduced in hERG1a using the QuikChange XL site-directed mutagenesis kit (Stratagene-Agilent, Stockport, UK) and subsequently confirmed by dye termination DNA sequencing.

HEK-293 cells (ECACC, European collection of cell cultures) were maintained in Dulbecco’s Minimum Essential Medium with Glutamax-1 (DMEM; Gibco, UK), supplemented with 10% Foetal Bovine Serum (FBS; UK), and 1% Penicillin/Streptomycin (P/S: Gibco, UK). HEK-293 cells stably expressing WT hERG-1a were treated in the same manner, except medium was also supplemented with 400 µg.ml^−1^ geneticin. Cells were plated onto 35 mm cell culture dishes (Corning, UK) and incubated at 37 °C for 24 hours before recording (from cells stably expressing hERG1a or transient transfections). CHO cells were maintained and treated in the same way as HEK-293 cells, except that the cells were maintained in Ham’s F-12K medium (Life Technologies). Cells were transiently transfected using polyethyleneimine (Alfa Aesar, Inc.) by combining channel plasmid DNA with enhanced green fluorescent protein DNA in a ratio of 1:5 (maximal plasmid content: 1.5 µg). Ratio of co-expressed subunit plasmid DNA was 1:1. Cells expressing enhanced green fluorescent protein (eGFP) were used for electrophysiology 16–24 hrs after transfection.

### Electrophysiology

Expressed hERG1a or 1b currents were recorded in the whole-cell or cell-attached configurations of the patch clamp technique. Cells were superfused (20 ml min^−1^) with a solution that contained (in mM): 140 NaCl, 4 KCl, 2 CaCl_2_, 1 MgCl_2_, 10 Glucose, 5 HEPES, and 5 MES (titrated to pH 7.4 with NaOH). MES was used as well as HEPES as MES acts as a buffer in the pH range 5.5–6.7 with a pK_a_ 6.1. The acidic external solution had the same composition as the above solution but titrated to the desired pH with HCl. Osmolarity of the external solution was approximately between 285–300 mOsm. Whole-cell electrodes fabricated from borosilicate glass were filled with a solution that contained (in mM): 130 KCl, 5 EGTA, 1 MgCl_2_, 10 HEPES, and 5 MgATP (titrated to pH7.2 with KOH), to give electrode resistances between 2.5 and 4.5 MΩ. The osmolarity of the pipette solution was ~270 mOsm. Single-channel experiments used the same solution composition for both external and pipette solutions (quartz electrodes were used throughout), which contained (in mM): 140 KCl, 2 CaCl_2_, 1 MgCl_2_, 5 HEPES, and 5 MES and titrated to either pH 7.4 or 6.3 with KOH. All salts were purchased from Sigma Aldrich, UK. Currents were recorded using an Axopatch 200 A amplifier (Axon Instruments). Currents were low-pass filtered at 1 kHz (8-pole Bessel, Frequency Devices) and data were acquired at 5–10 kHz using Pulse (HEKA). All recordings were made at room temperature (~22 °C). Series resistance and cell capacitance compensation was always performed, with series resistance values in the range of 2–10 MΩ and approximately 80% of the series resistance compensated. Membrane cell capacitance compensation values were in the range of 15–23 pF for HEK-293 cells and 11–18 pF for CHO cells.

### Data analysis

Whole-cell currents were analysed using Pulse (HEKA) and Origin (OriginLab). Single-channel activity was analysed using TAC (Bruxton, Seattle, USA), where channel openings were resolved using the 50% threshold technique to estimate event amplitudes and duration. Each transition was visually inspected before acceptance. Open- and closed-duration histograms were constructed using TacFit (Bruxton) and fitted with the sum of exponential probability functions using the maximum-likelihood method, with all bins being used for fitting. Correction for the filter rise time, together with the filter deadtime, dictated that openings shorter than 100 µs duration were underestimated. Data are presented as mean ± standard error of the mean (SEM). Statistical tests performed were chosen appropriately and two-tailed Student’s *t* test (paired or un-paired) or one/two-way analysis of variance (ANOVA) with a Bonferroni post-test were used. Statistical significance was assumed with *P* values of less than 0.05.

To determine the voltage-dependence of I_hERG_ activation, whole-cell tail currents from the standard current-voltage protocol were normalised to the peak hERG I_Tail_ and plotted against voltage. The plot was then fitted with a Boltzmann function of the form:

$$I=\frac{{I}_{max}}{1+exp(\frac{{V}_{0.5}-{V}_{m}}{k})}$$

where *I* is the peak I_hERG_ tail amplitude at test potential *V*_*m*_; *I*_*max*_ is the maximum I_hERG_ tail observed; *V*_*0.5*_ is the half-maximal voltage of I_hERG_ activation, and *k* is the slope of the fitted relationship.

Deactivation of hERG was determined by fitting I_Tail_ with a bi-exponential equation of the form:

$$y={A}_{s}.exp(\frac{-x}{{\tau }_{s}})+{A}_{f}.(\frac{-x}{{\tau }_{f}})+C$$

where *y* is the I_hERG_ amplitude at time x, τ_s_ and τ_f_ are the slow and the fast time constant of the two components of hERG I_Tail_ deactivation, A_s_ and A_f_ represent the total current fitted by the fast and the slow time-constants respectively, and C is any unfitted residual current.

Fractional reduction of hERG I_Tail_ by lowering the extracellular pH to either pH 6.3 was calculated using the following equation:

$${Fractional}\,Block=1-{I}_{(hERG(pH6.3orpH5.5))}/{I}_{(hERG(pH7.4))}$$

where *I*_*hERG (pH7.4)*_is the amplitude of the tail current in control and *I*_*hERG (ph6.3 or pH5.5)*_is the amplitude of I_Tail_ in the respective extracellular pH. Fractional block was plotted against the respective membrane potential.

The pK_a_ of these processes were measured using a dose-response curve with a variable Hill slope using GraphPad Prism (v7.0, Graphpad Inc.) of the form:

$$y={y}_{min}+\frac{{y}_{max}-\,{y}_{min}}{1+{10}^{(Logx0-x)p}}$$

where *y*_*min*_ and *y*_*max*_ are the minimum and maximum points of the curve respectively, Log*x*0 is the mid-point of the curve and *p* is the Hill slope of the curve.

Finally, to determine the pK_a_ of different processes, the percentage decrease of I_hERG_ was measured across the range of tested pH_e_ values, with the percentage decrease measured as

$$ \% \,Decrease=(1-\frac{{I}_{hERG(pH)}}{{I}_{hERG(control)}})\cdot 100$$

where *I*_*hERG (control)*_ is the process being measured (I_End pulse_, I_Tail_, τ_fast_ or τ_slow_) in control pH 7.4 and *I*_*hERG (pH)*_ is the process measured in a selected pH_e_.

### Modelling

Several sections of extracellular loops in the cryoEM structure of a hERG construct (PDB:5VA1)^24^ were modelled into the structure using Modeller v9.17^34^ (white sections of ribbon in Fig. 5a,b) and Procheck^35^ to assess model quality. The surface electrostatic potential was calculated using APBS^36^ following conversion of the loop-modelled cryoEM structure using the PDB2PQR server^37^.

## Supplementary information

Supplementary information

## Data Availability

Materials, data and associated protocols will be made available to readers upon reasonable request, without undue qualifications.
