# Supplementary material for: Identification of a proton sensor that regulates conductance and open time of single hERG channels
Source: Sci Rep. 2019 Dec 27;9:19825. doi: 10.1038/s41598-019-56081-y (PMC6934679; doi:10.1038/s41598-019-56081-y)
Supplement: Supplementary file 1 — Supplementary information [file 41598_2019_56081_MOESM1_ESM.pdf]

Identification of a proton sensor that regulates conductance and open time of single hERG channels

Stacey L. Wilson[1*], Christopher E. Dempsey[2], Jules C. Hancox[1+*]& Neil V. Marrion[1+]
1. School of Physiology, Pharmacology & Neuroscience; 2. School of Biochemistry, University of Bristol, Bristol, BS8 1TD, UK.

*Present address: Covance, Wooley Road, Alconbury, Huntingdon, Cambridgeshire, PE28 4HS, UK.
+ joint lead authors

***Corresponding Authors**
Jules C. Hancox, School of Physiology, Pharmacology & Neuroscience; University of Bristol, Bristol, BS8 1TD, UK. Phone: 44-1173312292 Fax: 44-1173312288; Email: jules.hancox@bristol.ac.uk

Neil V Marrion, School of Physiology, Pharmacology & Neuroscience; University of Bristol, Bristol, BS8 1TD, UK. Phone: 44-1173311401 Fax: 44-1173312288;Email: n.v.marrion@bristol.ac.uk

ONLINE SUPPLEMENTAL FIGURES

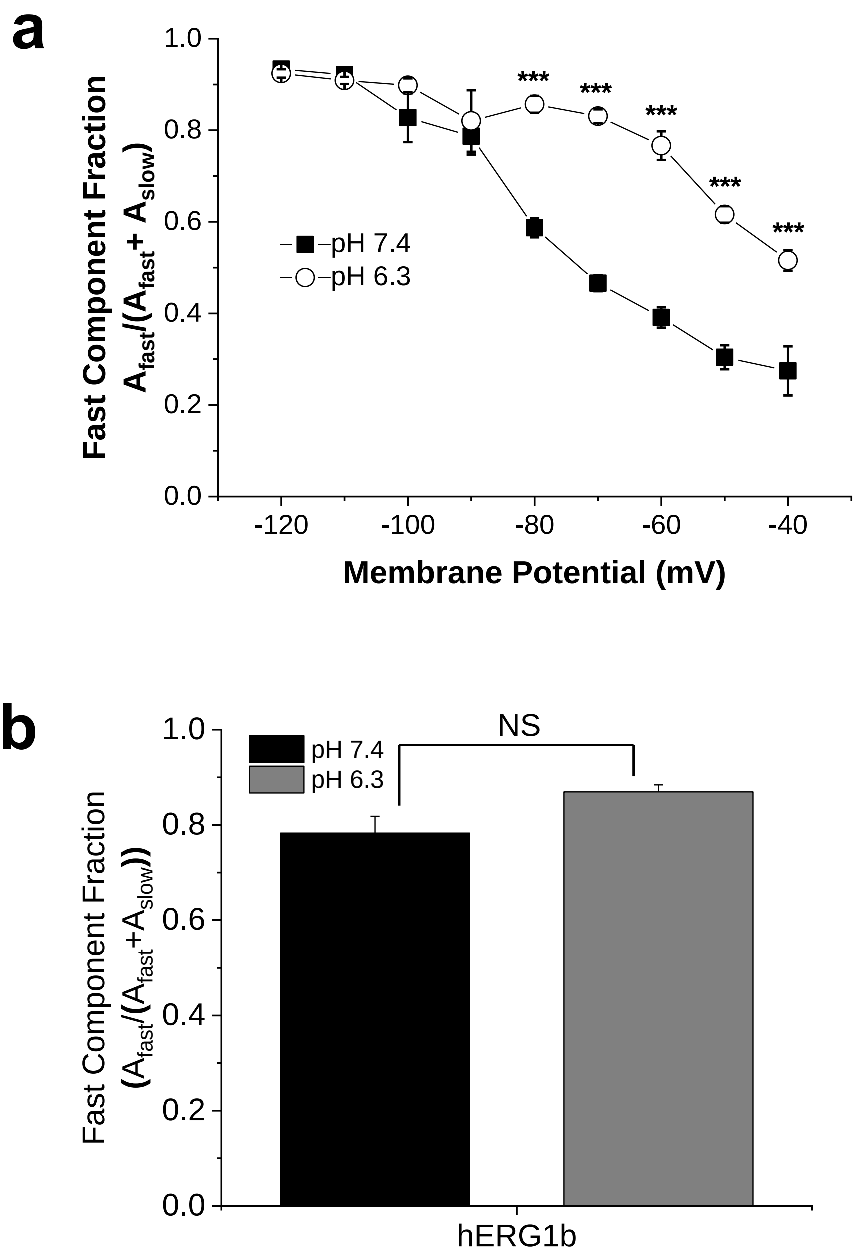

Supplementary Fig S1

(a) Contribution of fast and slow components of deactivation of hERG1a-mediated I_{hERG}. Deactivation was described by the sum of two exponential components, with time-constants plotted at pH_e 7.4 and 6.3 in Figure 1aiv of main paper. At pH 7.4 the contribution of the fast component of deactivation increased at progressively negative membrane potentials (filled circles). At pH 6.3 the contribution of the fast component of deactivation was increased between -40 and -80 mV, but not at more negative membrane potentials relative to that at pH 7.4 (*** $P < 0.001$ (2-way ANOVA with Bonferroni post-test; n=15 for pH 7.4 and 8 for pH 6.3 respectively)

(b) Bar chart showing the fraction of hERG1b deactivation described by the fast exponential component. Under the majority of hERG1b I_{hERG} deactivation at -40 mV was described by a fast exponential component at pH 7.4 (black bar), a contribution that did not change with extracellular acidosis (grey bar). ($P=0.052$ (paired t-test; n=5 for both pH 7.4 and pH 6.3). Corresponding deactivation time constant values are given in Figure 1biv of the main paper.

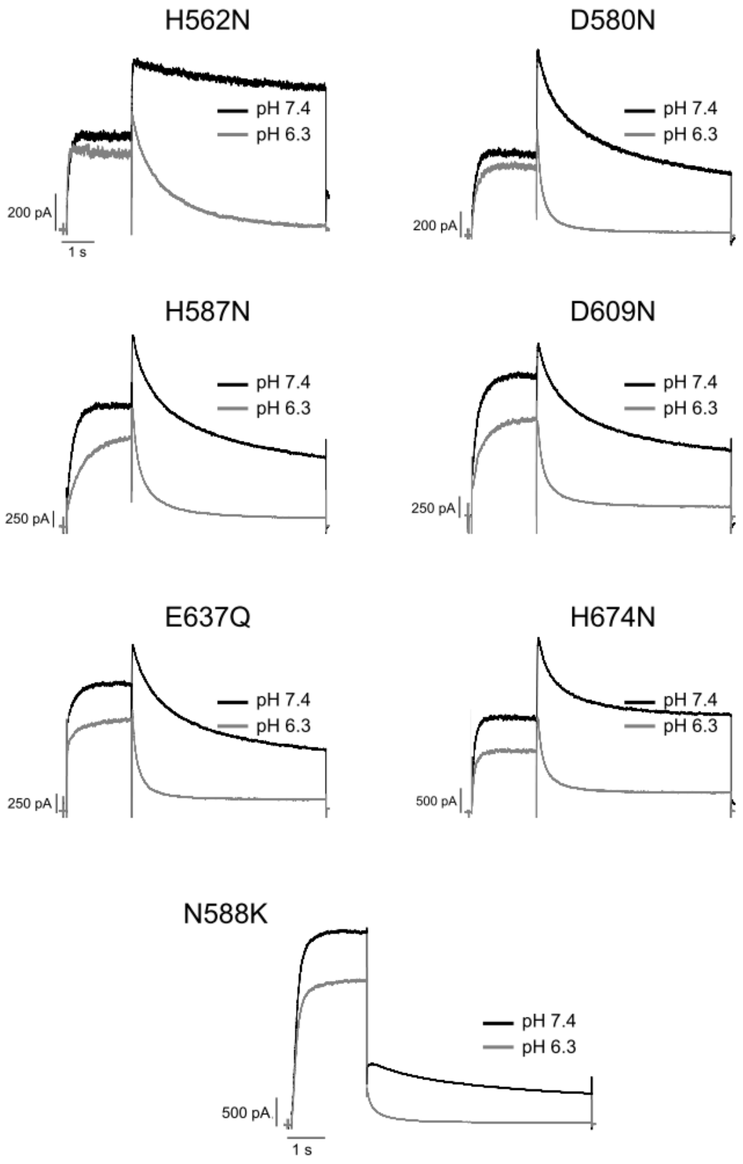

Supplementary Figure S2

Effect of extracellular acidosis on point mutants of hERG1a.
Representative traces are shown of membrane current evoked by a step depolarization to +20 mV, followed by repolarization to -40 mV (holding potential -80 mV) for the above stated mutations. Corresponding mean data are plotted in Fig 6 of the main paper.

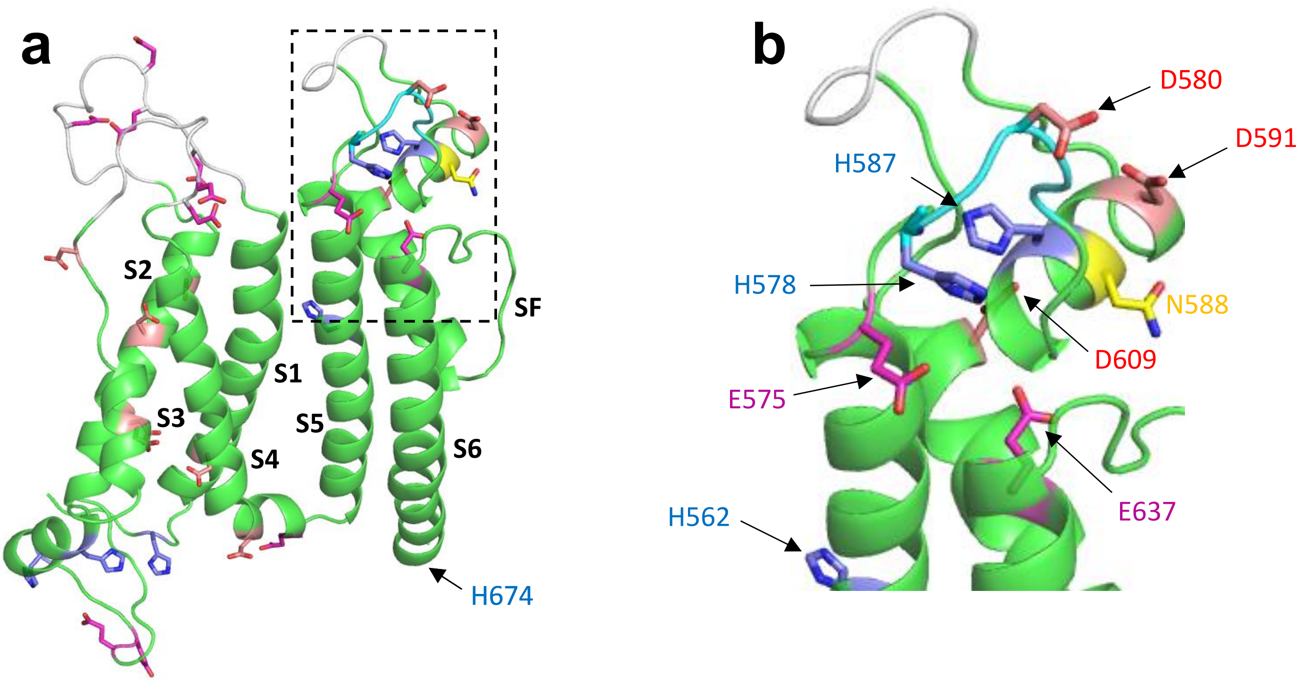

Supplementary Figure S3

Membrane domain subunit of the hERG cryoEM structure with missing density in the short loop H578 - R582 (pale blue backbone ribbon) modelled using Modeller v9.17 (https://salilab.org/modeller/) with a weak constraint to maintain E575 and H578 side chain β carbons within 6 angstroms. Other loops with missing density (white backbone ribbon) were modelled without constraints. **(a)** shows a single hERG subunit, whilst the region enclosed in the dashed box comprises most of the extracellular turret between helices S5 and S6 and is shown in **(b)**. Close approach of E575 and H578 side chains can be accommodated while maintaining allowed backbone phi-psi angles according to Ramachandran analysis using Procheck indicating that flexibility in this loop may allow close approaches of these side chains in hERG.
